# C-reactive protein is an independent predictor for hepatocellular carcinoma recurrence after liver transplantation

**DOI:** 10.1371/journal.pone.0216677

**Published:** 2019-05-29

**Authors:** Tobias Meischl, Susanne Rasoul-Rockenschaub, Georg Györi, Wolfgang Sieghart, Thomas Reiberger, Michael Trauner, Thomas Soliman, Gabriela Berlakovich, Matthias Pinter

**Affiliations:** 1 Division of Gastroenterology und Hepatology, Department of Medicine III, Medical University of Vienna, Vienna, Austria; 2 Liver Cancer (HCC) Study Group Vienna, Medical University of Vienna, Vienna, Austria; 3 Division of Transplantation, Department of Surgery, Medical University of Vienna, Vienna, Austria; 4 Vienna Hepatic Hemodynamic Lab, Medical University of Vienna, Vienna, Austria; University of Navarra School of Medicine and Center for Applied Medical Research (CIMA), SPAIN

## Abstract

**Background:**

Serum C-reactive protein (CRP) is a prognostic factor for overall survival (OS) and recurrence of hepatocellular carcinoma (HCC) in patients treated with resection or non-surgical treatment. Here, we investigated the association of elevated CRP (≥1 vs. <1 mg/dL) with (i) recurrence of HCC and (ii) OS after liver transplantation (LT).

**Methods:**

Adult HCC patients undergoing orthotopic deceased donor LT at the Medical University of Vienna between 1997 and 2014 were retrospectively analysed.

**Results:**

Among 216 patients included, 132 (61.1%) were transplanted within the Milan criteria and forty-two patients (19.4%) had microvascular invasion on explant histology. Seventy patients (32.4%) showed elevated CRP (≥ 1 mg/dL). On multivariate analysis, a CRP ≥ 1 mg/dL was an independent risk factor for HCC recurrence with a 5-year recurrence rate of 27.4% vs. 16.4% (HR 2.33; 95% CI 1.13–4.83; p = 0.022). OS was similar in patients with normal vs. elevated CRP levels.

**Conclusions:**

Elevated serum CRP is associated with HCC recurrence after LT and may be a marker for more aggressive tumor biology. Future studies should evaluate whether patients with elevated pre-transplant CRP levels benefit from closer monitoring for HCC recurrence.

## Introduction

HCC is the fifth most common cancer worldwide, the second most frequent cause of cancer-related death, and the most common cause of death in patients with liver cirrhosis.[1,2] The Barcelona Clinic Liver Cancer (BCLC) classification is recommended for staging and treatment allocation and stratifies patients into five categories according to their liver function, performance status, and tumor characteristics.[3,4] For selected patients, liver transplantation (LT) is the treatment of choice since it cures both HCC and underlying liver cirrhosis. After the introduction of the Milan criteria[5]–aiming to reduce the rate of recurrence in immunosuppressed patients by minimizing the risk of micrometastases at the time of transplantation–outcome has greatly improved and excellent survival rates (over 70% after five years), similar to those of patients transplanted for non-malignant indications, have been achieved.[5,6] More liberal criteria for selection of HCC for LT have been suggested but there is significant controversy on their clinical applicability. [7–9]

HCC recurrence after transplantation is a major problem even in well-selected patients. Since patients need to remain on immunosuppression HCC recurrence usually results in a dismal prognosis.[10,11] However, early detection of tumor recurrence may be key to improve the outcome of these patients.

C-reactive protein (CRP) plays an important role in the development and/or prognosis of various types of cancer, such as esophageal squamous cell carcinoma, cervical cancer, and non-small cell lung cancer.[12–14] In HCC, CRP predicted poor overall survival and recurrence rates after hepatic resection[15,16] and was related with a poor outcome after living-donor liver transplantation.[17–19] We previously showed that CRP was independently associated with OS in a large cohort of non-surgical HCC patients and validated our results in an external cohort.[20] In the present study, we investigated the prognostic value of pre-transplant serum CRP in HCC patients undergoing orthotopic LT.

## Materials and methods

### Patients

We included patients at the age of 18 years or higher (at time of LT) who were diagnosed with HCC and underwent orthotopic liver transplantation at the Medical University of Vienna between May 1997 and August 2014. Patients with missing pre-transplant CRP or missing follow-up data were excluded.

In general, patients fulfilling the Milan criteria[5] were candidates for LT. Patients exceeding the Milan criteria, but fulfilling expanded criteria (up-to-seven[7] or UCSF criteria[21]), and/or who have undergone successful downstaging could also be considered for LT. Absolute contraindications included extrahepatic metastases, severe cardiac and/or pulmonary diseases and severe pulmonary hypertension (mean pulmonary arterial pressure >45 mm Hg), ongoing alcohol abuse, ongoing extrahepatic malignancies and ongoing sepsis. Relative contraindications included morbid obesity, advanced age and persistent non-adherence.[22]

This retrospective analysis was approved by the local human research ethics committee of the Medical University of Vienna (reference number 1759/2015, approved on 13^th^ of November, 2015). Written, informed consent was not obtained from the patients included in this study as the study is a retrospective analysis of anonymised patient data.

### Data collection

All data were retrospectively collected from the centre’s transplant database. The date of liver transplantation was considered the baseline of this study.

HCC was diagnosed by radiological imaging—multiphase computed tomography (CT) and/or contrast enhanced magnetic resonance imaging (MRI)—or by pre-transplant biopsy. Any pathological analysis was performed by a senior liver pathologist and tumor grading was staged according to Edmondson and Steiner[23]. All blood parameters recorded for this study, including CRP levels, prothrombin time, bilirubin, and albumin were taken at admission for LT—in the ISO-certified laboratory of the Medical University of Vienna. Only alpha-1-fetoprotein (AFP) was mostly determined earlier than the date of LT.

Child-Pugh score was recorded to describe liver function. Tumor stage was recorded according to the BCLC classification.[3,4,24]

All LT procedures in this study were performed with grafts from brain death deceased donors by specialized transplant surgeons at the Medical University of Vienna. After LT, routine follow-up consisted of clinical and radiological examination every 6 months after transplantation.

### Statistical analyses

In a previous study of HCC patients treated with non-surgical therapy, we assessed the CRP cut-off by regression spline analysis and found <1/≥1 mg/dL to be the optimal cut-off to predict survival.[20] This cut-off was validated in an independent external cohort[20], as well as in several other studies of patients with HCC [15–17] and other malignancies[12,14]. Therefore, we used this cut-off to categorize patients into two subgroups (CRP <1 mg/dL and ≥1 mg/dL).

Baseline clinical data and tumor characteristics of the overall study population and the subgroups CRP <1.0 mg/dL vs. ≥1.0 mg/dL) were presented using descriptive statistics. Differences between the two subgroups were assessed by Χ^2^-test. Time to recurrence (TTR) was defined as the time from the date of LT until the date of recurrence of HCC diagnosed either radiologically or histologically. Patients without documented recurrence were censored at the date of the last radiological follow-up examination. Overall survival (OS) was defined as the time from the date of LT until the date of death. Patients who were still alive at March 31, 2018 (end of follow-up) were censored at the time of last contact. Survival curves were calculated by the Kaplan-Meier method and compared by means of the log rank test (univariate analysis). Variables that reached a p-value of 0.1 or less on univariate analysis were entered into multivariate analysis using a Cox proportional hazards model.

A p-value < 0.05 was considered significant. Statistical analyses were performed using SPSS version 25.0 (Chicago, IL, USA).

## Results

### Patient characteristics

**Fig 1:**
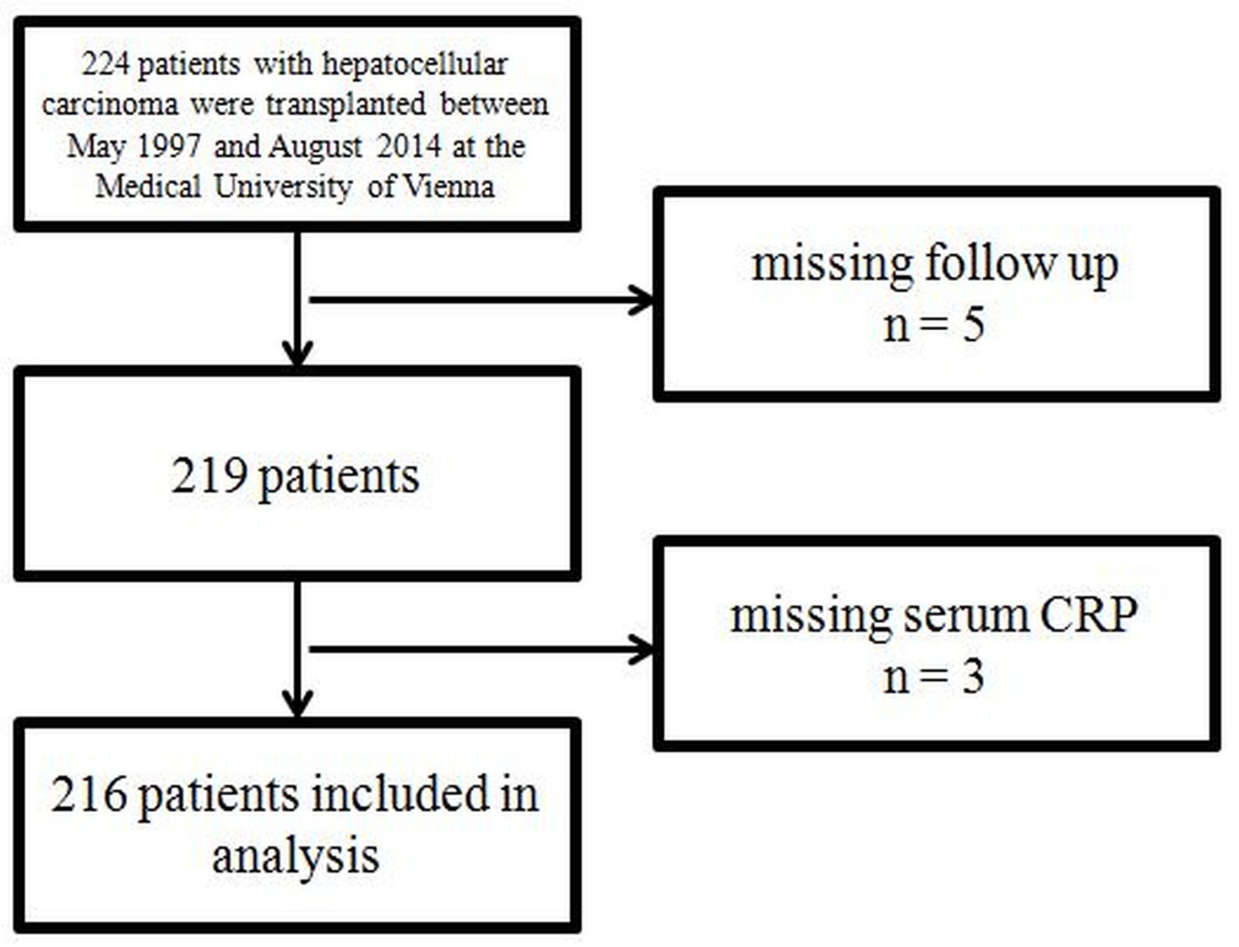
Patient flow chart.

Of 224 patients transplanted for HCC, 5 patients were excluded because of missing follow-up data and 3 patients because of missing serum CRP (Fig 1). Table 1 displays the patient characteristics of the 216 eligible patients, all of whom received deceased donor liver transplantation. Median duration of follow-up was 95.9 months.

**Table 1:** Patient characteristics.

		overall study population		CRP < 1 mg/dL		CRP ≥ 1 mg/dL		Χ^2^-test (p value)
		N	%	N	%	N	%	
Total		216	100	146	67.6	70	32.4	
Age (years)	≥ 65	26	12.0	17	11.6	9	12.9	0.798
	< 65	190	88.0	129	88.4	61	87.1	
Sex	Male	189	87.5	125	85.6	64	91.4	0.227
	Female	27	12.5	21	14.4	6	8.6	
Etiology	HCV	99	45.8	73	50.0	26	37.1	0.089
	Alcohol	67	31.0	44	30.1	23	32.9	
	HBV	23	10.3	16	11.0	7	10.0	
	Unknown/other	27	12.5	13	8.9	14	20.0	
Child Pugh class*	A (score 5 or 6)	57	26.4	49	33.6	8	11.4	< 0.001
	B (score 7–9)	89	41.2	62	42.5	27	38.6	
	C (score 10 or higher)	48	22.2	23	15.8	25	35.7	
Tumor size (cm) - pathology	≤ 3	140	68.4	99	67.8	41	58.6	0.134
	> 3 and ≤ 5	52	24.1	35	24.0	17	24.3	
	> 5	24	11.1	12	8.2	12	17.1	
Number of nodules - pathology	1	98	45.4	71	48.6	27	38.6	0.360
	2	58	26.9	34	23.3	24	34.3	
	3	39	18.1	27	18.5	12	17.1	
	> 3	21	9.7	14	9.6	7	10.0	
Vascular invasion - pathology	Absent	174	80.6	120	82.2	54	77.1	0.380
	Present	42	19.4	26	17.8	16	22.9	
AFP pre-LT (ng/mL)^x^	< 200	172	79.6	120	82.2	52	74.3	0.564
	200–400	5	2.3	4	2.7	1	1.4	
	400–1000	6	2.8	4	2.7	2	2.9	
	> 1000	6	2.8	5	3.4	1	1.4	
CRP (mg/dL)	< 1	146	67.6	146	100	0	0.0	-
	≥ 1	70	32.4	0	0.0	70	100	
Treatment prior to LT^†^	No Treatment	76	35.2	48	32.9	28	40.0	0.119
	Any Treatment	132	61.1 (100)	97	66.4 (100)	35	50.0 (100)	
	TACE	43	19.9 (32.6)	32	21.9 (33.0)	11	15.7 (31.4)	
	PEI	49	22.7 (37.1)	37	25.3 (38.1)	12	17.1 (34.3)	
	RFA	15	6.9 (11.4)	13	8.9 (13.4)	2	2.9 (5.7)	
	Other/combined	25	11.6 (18.9)	15	10.3 (15.5)	10	14.3 (28.6)	
Milan in/out (as by explant pathology)	Milan in	132	61.1	93	63.7	39	55.7	0.260
	Milan out	84	38.9	53	36.3	31	44.3	
Tumor grade (Edmondson & Steiner classification)^‡^	G1	25	11.6	16	11.0	9	12.9	0.114
	G2	144	66.7	100	68.5	44	62.9	
	G3	16	7.4	7	4.8	9	12.9	

Abbreviations: AFP, Alpha-Fetoprotein; CRP, C-reactive protein; HBV, Hepatitis B Virus; HCV, Hepatitis C Virus; LT, liver transplantation

*Missing, n = 22 (10.2%)

^x^Missing, n = 27 (12.5%)

^†^Missing, n = 22 (10.2%)

^‡^Missing, n = 31 (14.4%)

One-hundred and eighty-nine subjects (87.5%) were male and 27 (12.5%) were female. According to explant histology, 132 patients (61.1%) were within Milan criteria. Forty-two patients (19.4%) had vascular invasion. Median serum AFP level was 8.20 IU/mL and 70 patients (32.4%) had a serum CRP level of 1mg/dL or higher at the time of transplantation. Patients with CRP ≥1 mg/dL had a higher Child-Pugh class (p < 0.001). All other variables were distributed equally between the two subgroups.

### Recurrence and survival

Of 216 patients included, 35 (16.2%) had recurrence of HCC after LT. Median time to recurrence was 21.2 months. Recurrence occurred between 2.6 months and 182.8 months after LT.

**Fig 2:**
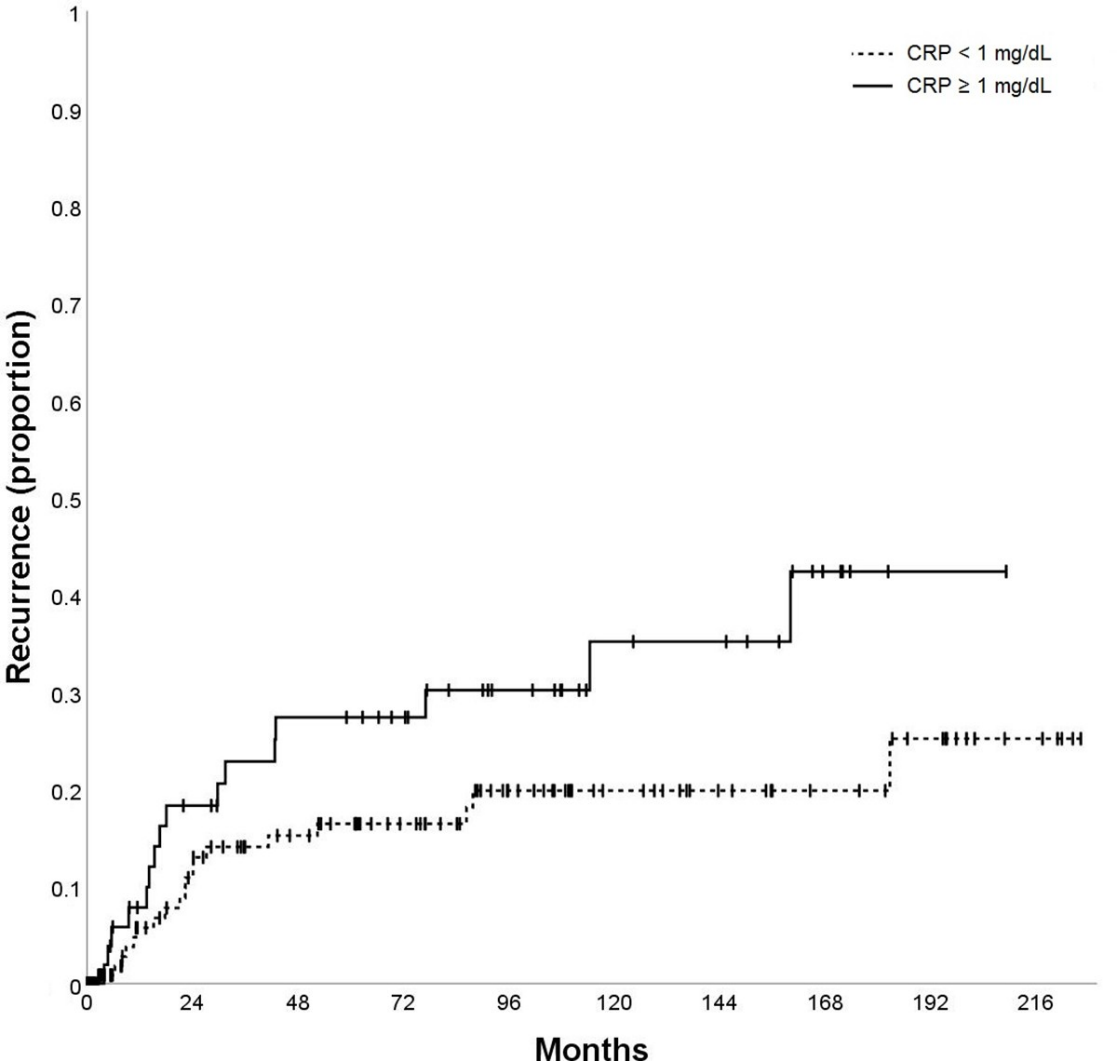
Kaplan-Meier curve according to serum C-reactive protein (CRP) level: Time to recurrence (TTR) according to CRP ≥ 1 mg/dL vs. CRP < 1 mg/dL.

Patients with CRP ≥ 1 mg/dL had higher recurrence rates than patients with CRP <1 mg/dL (5-year recurrence rate 27.4% vs. 16.4%; p = 0.055) (Table 2; Fig 2). Of 70 patients with CRP ≥ 1 mg/dL, 39 (55.7%) patients were within the Milan criteria and 31 (44.3%) patients were beyond Milan on explant histology.

**Table 2:** Univariate analysis of time to recurrence.

Variable		N	year				p value
			1	3	5	10	
Overall study population		216	6.4%	16.9%	20.0%	24.9%	-
Age	≥ 65	26	5.3%	16.1%	22.1%	22.1%	0.871
	< 65	190	6.6%	17.0%	19.7%	25.4%	
Sex	male	189	6.6%	15.4%	19.0%	24.6%	0.665
	female	27	5.0%	27.3%	27.3%	27.3%	
Child-Pugh Class	A	57	11.6%	26.6%	29.3%	29.3%	0.355
	B	89	5.8%	18.2%	21.6%	23.7%	
	C	48	2.8%	5.7%	9.3%	22.2%	
Tumor size (cm)—pathology	≤ 3	140	7.1%	15.0%	18.0%	19.8%	0.053
	> 3 and ≤ 5	52	5.2%	19.2%	25.3%	37.8%	
	> 5	24	23.3%	51.7%	51.7%	51.7%	
Number of nodules—pathology	1	98	1.5%	6.3%	12.0%	24.5%	0.005
	2–3	97	11.8%	27.3%	29.6%	38.2%	
	> 3	21	27.8%	35.8%	35.8%	35.8%	
Vascular invasion—pathology	absent	174	5.7%	14.4%	16.6%	21.2%	0.001
	present	42	16.8%	35.7%	54.6%	54.6%	
Milan—pathology	in	132	4.4%	11.6%	14.6%	18.0%	0.004
	out	84	12.8%	28.6%	35.1%	43.2%	
Tumor grade—pathology	G1	25	5.9%	17.6%	23.5%	23.5%	0.846
	G2	144	7.4%	20.3%	22.5%	28.2%	
	G3	16	8.3%	18.5%	18.5%	18.5%	
AFP pre-LT (ng/mL)	< 200	172	4.8%	13.3%	15.8%	21.7%	0.002
	≥ 200	17	21.7%	54.3%	54.3%	54.3%	
CRP pre-LT (mg/dL)	< 1	146	5.7%	14.0%	16.4%	19.8%	0.055
	≥ 1	70	7.8%	22.8%	27.4%	35.1%	

Abbreviations: AFP, Alpha-Fetoprotein; CRP, C-reactive protein; HBV, Hepatitis B Virus; HCV, Hepatitis C Virus; LT, liver transplantation

**Fig 3:**
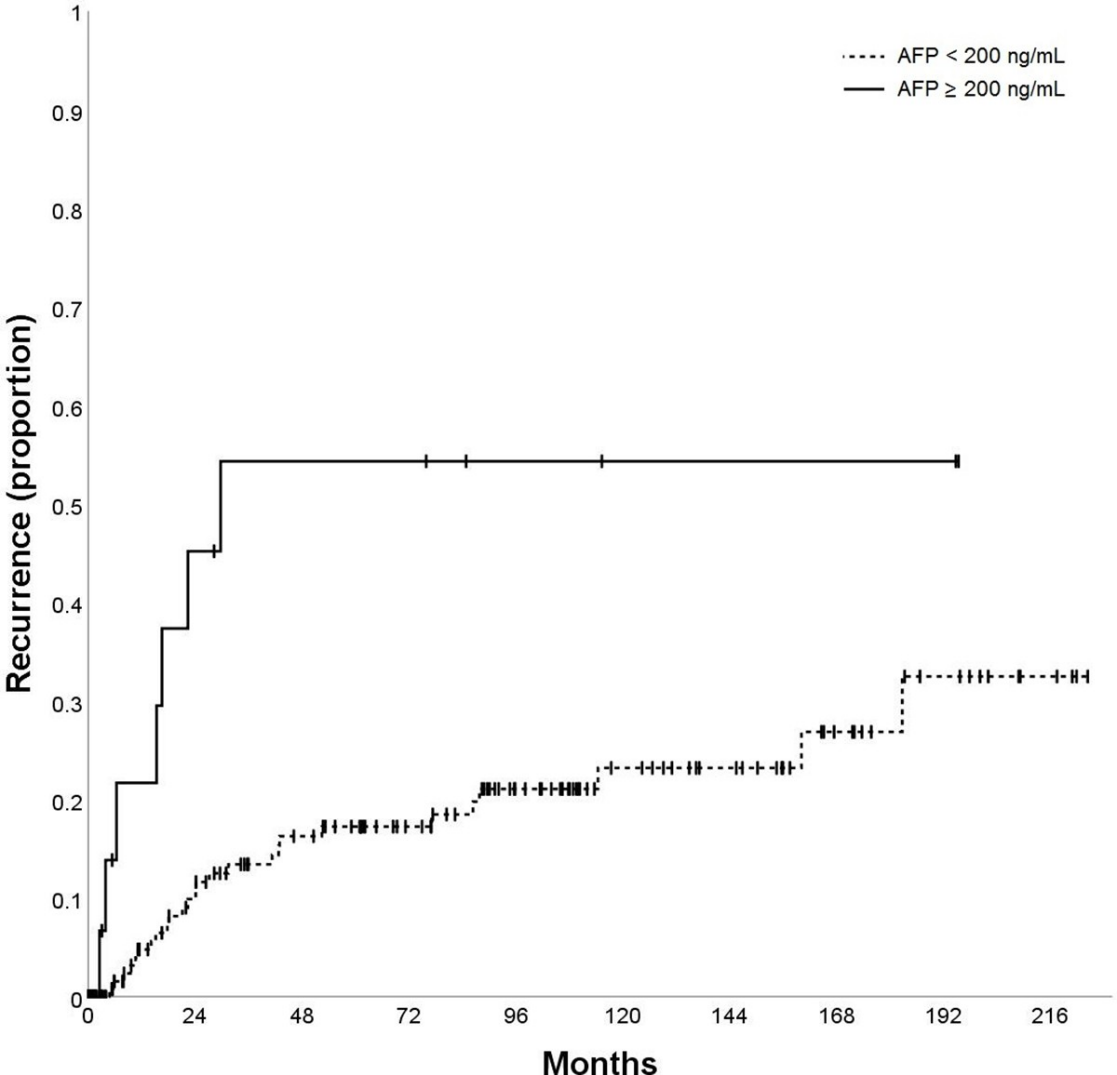
Time to recurrence (TTR) according to baseline alpha-fetoprotein (AFP).

**Fig 4:**
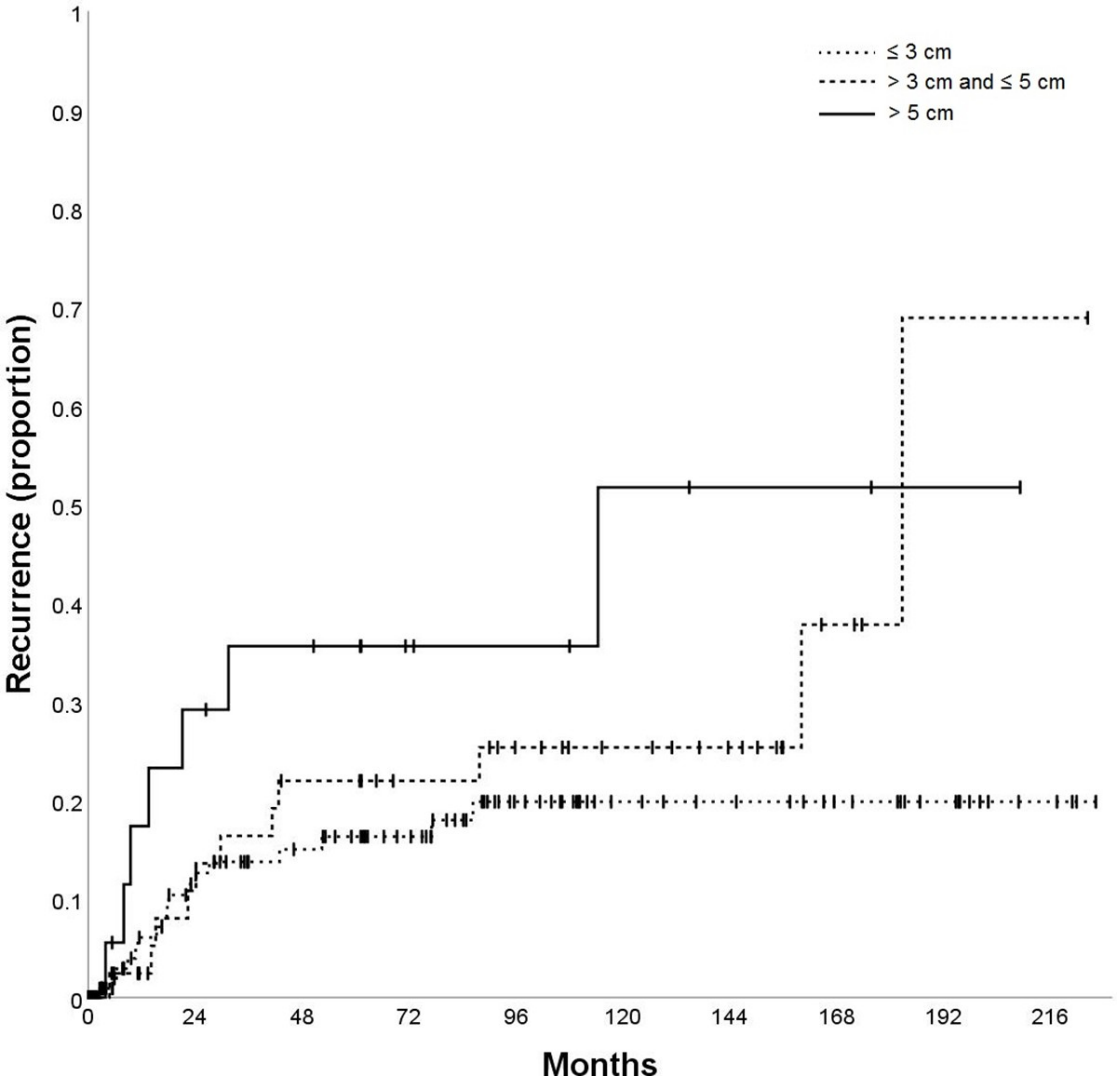
Time to recurrence (TTR) according to tumor size.

**Fig 5:**
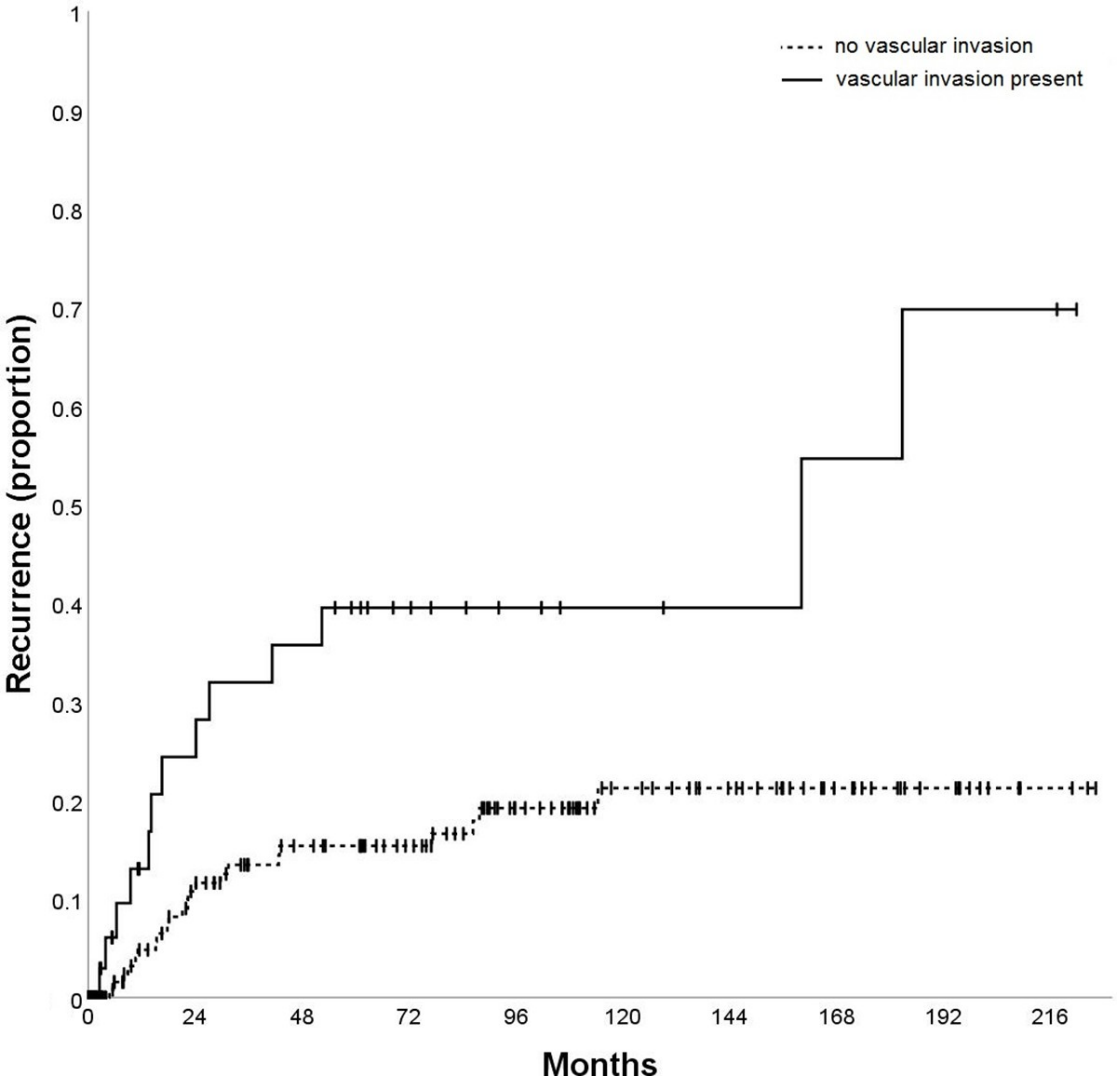
Time to recurrence (TTR) according to vascular invasion.

**Fig 6:**
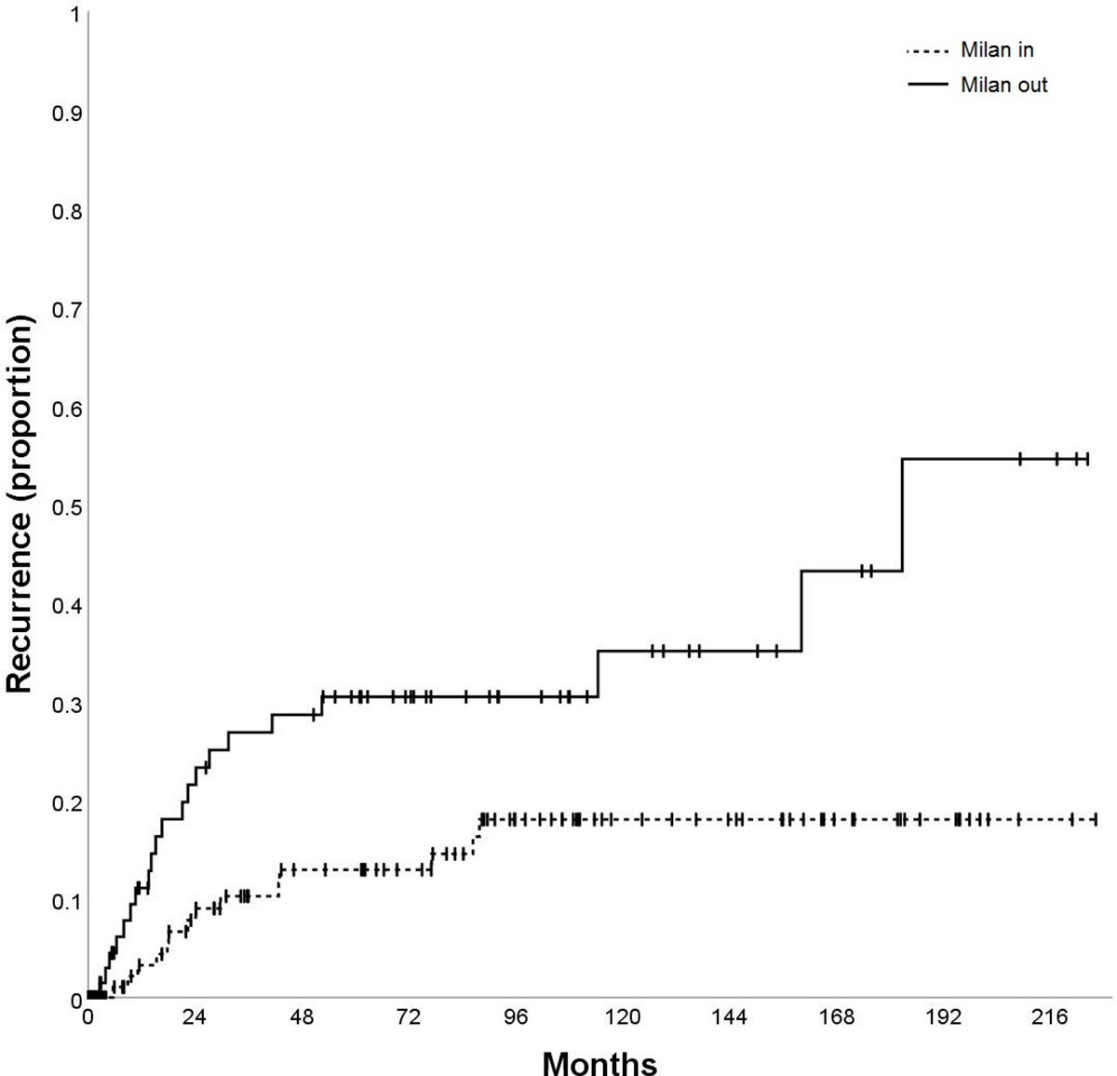
Time to recurrence (TTR) according to Milan status.

The variables AFP (p = 0.002; Fig 3), tumor size (p = 0.040; Fig 4), vascular invasion (p = 0.001; Fig 5), Milan in/out (p = 0.002; Fig 6), and number of nodules (p = 0.003) were also significantly associated with HCC recurrence (Table 2). Median time-to-recurrence (TTR) could not be calculated in the subgroups due to the low number of events.

All variables that reached a p-value of 0.100 or less were entered in the multivariate analysis model for TTR. The variable “Milan in/out” was not entered as this variable is directly derived from the variables "number of nodules", "tumor size", and "vascular invasion", all of which were included in the multivariate analysis separately. Serum CRP was an independent predictor for recurrence in multivariate analysis with a hazard ratio (HR) of 2.23 (95% CI 1.09–4.56; p = 0.028). Tumor size (p = 0.038), vascular invasion (p = 0.011), and AFP (p = 0.003) were independently associated with recurrence as well (Table 3).

**Table 3:** Multivariate analysis of time to recurrence.

Variable		N	HR	95% CI	P value (Cox regression)
Number of nodules	1	98	1.00	-	0.192
	2–3	97	2.04	0.90–4.61	
	> 3	21	2.44	0.69–8.61	
Largest tumor nodule (cm)	≤ 3	140	1.00	-	0.038
	> 3 and ≤ 5	52	1.70	0.77–3.75	
	> 5	24	3.58	1.33–9.67	
Vascular invasion	Absent	174	1.00	-	0.011
	Present	42	2.79	1.26–6.17	
AFP pre-LT (ng/mL)	< 200	172	1.00	-	0.003
	≥ 200	17	3.97	1.60–9.84	
CRP pre-LT (mg/dL)	< 1	146	1.00	-	0.028
	≥ 1	70	2.23	1.09–4.56	

Abbreviations: AFP, Alpha-Fetoprotein; CRP, C-reactive protein; LT, liver transplantation

**Fig 7:**
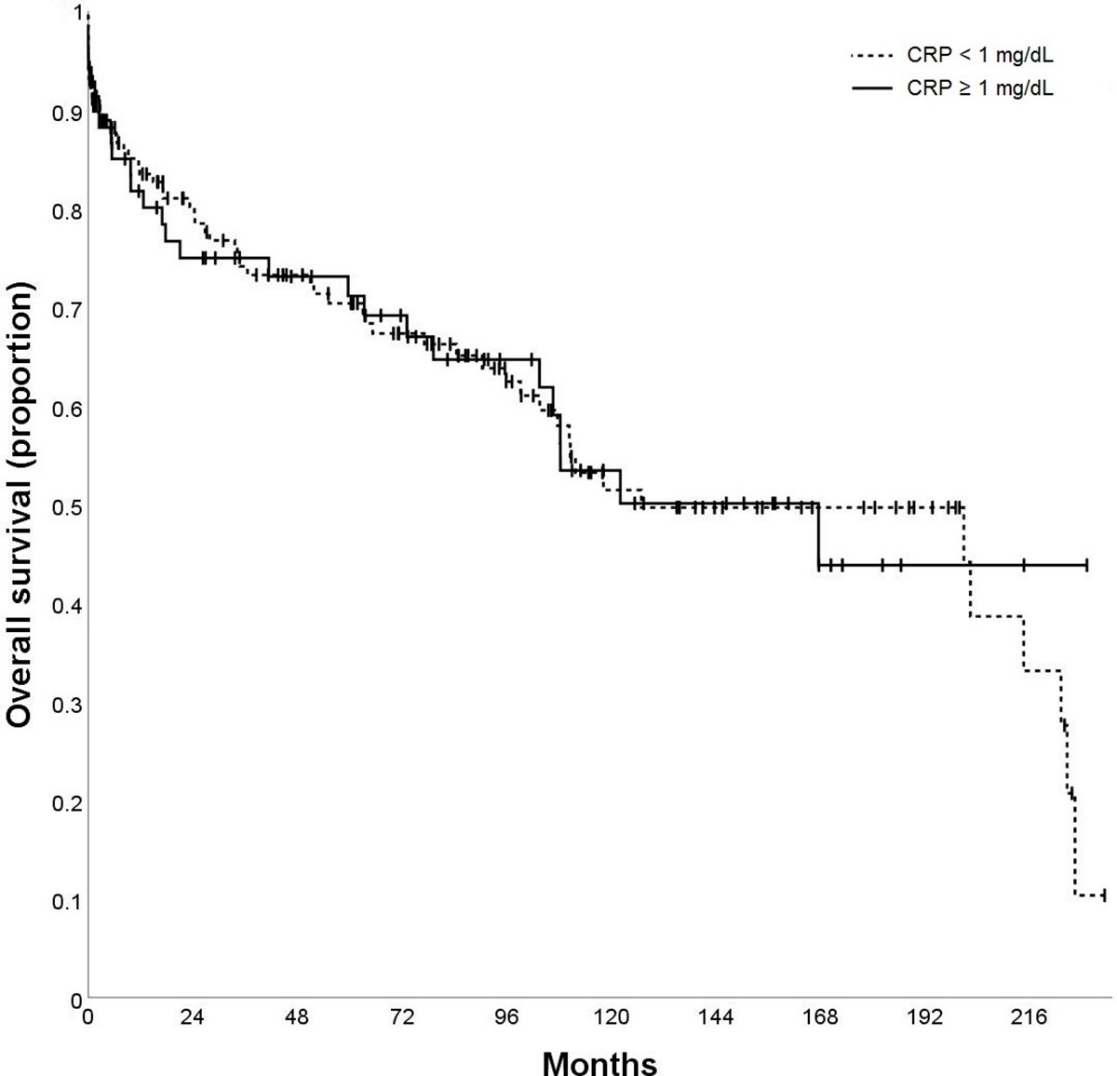
Kaplan-Meier curve according to serum C-reactive protein (CRP) level: Overall survival (OS) according to CRP ≥ 1 mg/dL vs. CRP < 1 mg/dL.

In total, 86 (39.8%) patients died during follow-up. Median OS of the total study population was 134.7 months with a 5-year survival rate of 70.0%. In univariate analysis, no difference of OS was observed in patients with CRP ≥ 1 mg/dl vs. CRP <1 mg/dl (p = 0.909; Table 4; Fig 7).

**Table 4:** Univariate analysis—overall survival.

Variable		N	Med.	95% CI	Survival rate				P value
					1 year	3 years	5 years	10years	
Overall study population		216	126.9	60.1–193.8	82.4%	73.8%	70.0%	50.9%	-
Age (years)	≥ 65	26	108.4	35.7–181.1	73.1%	61.3%	56.6%	49.5%	0.159
	< 65	190	126.9	43.4–210.4	84.3%	76.4%	72.7%	52.5%	
Sex	Male	189	167.6	108.4–226.8	84.3%	77.0%	72.6%	54.6%	0.057
	Female	27	84.5	4.7–164.4	73.6%	57.4%	57.4%	36.1%	
Child-Pugh class*	A	57	103.6	18.4–188.9	83.5%	74.7%	70.0%	45.6%	0.558
	B	89	126.9	56.0–197.8	87.0%	75.1%	72.4%	53.2%	
	C	48	–		84.5%	79.7%	73.7%	61.7%	
Tumor size (cm)—pathology	≤ 3	140	200.9	120.8–281.1	82.6%	75.4%	72.3%	55.1%	0.424
	> 3 and ≤ 5	52	108.3	68.8–147.9	78.3%	67.4%	65.0%	45.0%	
	> 5	24	73.1	8.8–137.5	80.5%	68.1%	55.1%	31.5%	
Number of nodules—pathology*	1	98	200.9	77.1–324.7	85.0%	81.2%	79.8%	52.9%	0.258
	2–3	97	108.3	61.9–154.8	78.1%	65.6%	58.4%	44.1%	
	> 3	21	–		77.6%	70.6%	70.6%	70.6%	
Vascular invasion—pathology	Present	42	223.2	10.9–435.5	75.6%	56.8%	26.7%	26.7%	0.348
	Absent	174	167.6	96.1–239.1	83.2%	76.8%	73.7%	51.7%	
Milan in/out (pathology)	in	132	200.9	88.6–313.2	83.3%	78.6%	76.5%	54.5%	0.199
	out	84	108.3	57.3–159.4	79.5%	64.6%	58.3%	42.5%	
Tumor grade*	G1	25	108.3	9.3–207.3	66.9%	58.5%	58.5%	37.3%	0.164
	G2	144	200.9	114.3–287.5	89.6%	78.3%	72.6%	57.3%	
	G3	16	–		80.2%	80.2%	80.2%	53.5%	
AFP (ng/mL)*	< 200	172	122.1	64.4–179.9	81.2%	73.7%	70.0%	51.2%	0.821
	>200	17	224.6	–	100%	65.6%	56.3%	56.3%	
CRP pre-LT (mg/dL)	≥ 1	70	167.6	84.6–250.6	83.5%	74.1%	70.4%	51.4%	0.909
	< 1	146	126.9	47.6–206.3	81.7%	75.0%	71.1%	53.4%	

Abbreviations: AFP, Alpha-1-Fetoprotein; CRP, C-reactive protein; LT, liver transplantation.

* median survival and/or 95%-confidence interval could not be calculated due to insufficient number of events in this subgroup

### Subgroup analysis of patients with recurrence of HCC

In the 35 patients who experienced HCC recurrence (n = 13 intrahepatic, n = 22 extrahepatic), 21 patients (60%) died during follow-up and median OS was 14.6 (95% CI 5.5–23.7) months, calculated from the date of detection of recurrence. OS was impacted by the type of treatment after recurrence: median OS was 72.0 months (95% CI 3.9–140.1) in the 12 patients treated with curative intent (e.g. resection, local ablation) for intrahepatic recurrence vs. 10.7 months (95% CI 0.1–21.3) in the 21 patients who received palliative treatment (e.g. TACE, systemic treatment, best supportive care) (p = 0.003), of which 16 patients had extrahepatic recurrence. Two patients in whom recurrence was diagnosed in autopsy were excluded from this analysis.

In the overall study population, patients with recurrence of HCC had a significantly shorter survival (calculated from the date of liver transplantation) with a median OS of 41.5 months (95% CI 7.0–76.0) for patients who experienced recurrence (n = 35) vs. 200.9 months (95% CI 119.4–282.5) for those without recurrence (n = 181; p = 0.003).

Detailed information on the pattern of recurrence is provided in a supporting table (S1 Table).

## Discussion

In the present study, we found that serum CRP of 1 mg/dL or higher was associated with higher tumor recurrence in patients who underwent deceased donor LT for HCC. Previous studies have reported that elevated CRP levels independently predicted poor outcome in HCC patients undergoing living donor LT.[17–19,25] Elevated CRP levels are also associated with poor outcome in non-transplanted HCC patients, as persistently elevated CRP before and after treatment correlated with poor OS in HCC patients mainly treated with loco-regional therapies.[26] CRP levels were also incorporated in prognostic scores developed for HCC patients (e.g. Glasgow prognostic score).[27–29]

We have previously demonstrated that serum CRP was associated with more aggressive tumor characteristics in non-surgical HCC patients and a prognostic factor in patients both with and without clinically overt infection.[20] Elevated CRP levels also indicate a higher risk of complications and mortality in patients with liver cirrhosis (without HCC).[30] Given that HCC usually develops in patients with cirrhosis[2,31], elevated CRP may reflect both more aggressive tumor behaviour as well as an increased risk of death from complications of liver cirrhosis.[2,20,30]

Since first proposed by Virchow in 1863, the connection between inflammation and malignancies has been well established.[32] CRP seems to play an important role in the development and/or prognosis of various types of cancer, such as esophageal squamous cell carcinoma, cervical cancer and non-small cell lung cancer.[12–14] Yet, the mechanistic role of CRP in HCC and in other types of cancer remains largely unclear. The question of whether aggressive tumor behaviour leads to a prognostically unfavourable inflammatory response and/or systemic or hepatic inflammation *per se* drives tumor progression needs further exploration.[20]

Recent studies proposed that CRP is mechanistically linked to hepatocarcinogenesis since upon interleukin-1 (IL-1) stimulation, EGFR-recruited liver macrophages induce interleukin-6 (IL-6).[33] IL-6 not only stimulates CRP production in hepatocytes[34,35] but also triggers hepatocyte proliferation and promotes development of HCC.[33,36,37] Notably, the presence of EGFR-positive liver macrophages in HCC patients was associated with poor survival.[33]

Other inflammatory markers such as the neutrophil-lymphocyte ratio (NLR) have been correlated to a poor prognosis in HCC patients treated with resection[38–40], liver transplantation[17,41,42], transarterial chemo-embolisation[43,44] or systemic therapy[45–48], suggesting that an “inflammatory phenotype” of HCC is associated with more aggressive tumor biology[49] and worse prognosis[50,51].

In contrast to the results of other studies[17–19,25], CRP was only associated with recurrence but not with overall survival of HCC patients undergoing liver transplantation. We want to acknowledge some differences to these studies,[17–19,25] which may have influenced the results at least to some degree, including the different geographical region (European vs. Asian cohort), use of different grafts for LT (living donor vs. deceased donor), and finally the number of patients which is higher in our study. Importantly, about one third of the patients with HCC recurrence in our study were amenable to curative treatment which resulted in an excellent outcome (median OS of 72 months). This could have prevented an association of elevated CRP with poorer survival despite the higher recurrence rate. The favourable outcome of patients whose recurrent HCC was treated with curative intent underlines the importance of early detection of HCC recurrence after LT that allows the use of potentially curative treatment options. Elevated pre-transplant CRP could be a useful parameter to select patients at higher risk of HCC recurrence.

The main limitations of this study are its retrospective design including potential biases and the limited number of patients in some subgroups.

In conclusion, serum CRP may represent a valuable surrogate parameter for a more aggressive tumor biology. Pre-transplant CRP levels could thus identify patients at higher risk for HCC recurrence after LT. Early detection of HCC recurrence may allow for curative treatment options and therefore may improve the outcome of these patients. Future studies should evaluate if closer HCC monitoring intervals in patients with elevated pre-LT CRP levels can improve their outcome and survival.

## Supporting information

S1 Table Pattern of recurrence. (DOCX)

S1 File Minimal data set. (XLSX)
