# Supplementary material for: C-reactive protein is an independent predictor for hepatocellular carcinoma recurrence after liver transplantation
Source: PLoS One. 2019 May 29;14(5):e0216677. doi: 10.1371/journal.pone.0216677 (PMC6541257; doi:10.1371/journal.pone.0216677)
Supplement: S1 Table — (DOCX) [file pone.0216677.s001.docx]

**S1 Table. Pattern of recurrence.**

| Recurrence (at time of detection of recurrence) | Overall study population | | CRP ≥ 1 mg/dL | | CRP < 1 mg/dL | |
| --- | --- | --- | --- | --- | --- | --- |
|  | N | % | N | % | N | % |
| Patients with recurrence | 35 | 100 | 16 | 45.7 | 19 | 54.3 |
| Intrahepatic | 13 | 37.1 (100) | 7 | 20.0 (53.8) | 6 | 17.1 (46.2) |
| Milan in | 7 | 20.0 (53.8) | 4 | 11.4 (30.8) | 3 | 8.6 (23.1) |
| Milan out | 6 | 17.1 (46.2) | 3 | 8.6 (23.1) | 3 | 8.6 (23.1) |
| Extrahepatic | 22 | 62.9 (100) | 9 | 25.7 (40.9) | 13 | 37.1 (59.1) |
| One extrahepatic organ | 11 | 31.4 (50) | 5 | 14.3 (22.7) | 6 | 17.1 (27.3) |
| Bone | 5 | 14.3 (22.7) | 2 | 5.7 (9.1) | 3 | 8.6 (13.6) |
| Peritoneum | 3 | 8.6 (13.6) | 1 | 2.9 (4.5) | 2 | 5.7 (9.1) |
| Lung | 1 | 2.9 (4.5) | 0 | 0 (0) | 1 | 2.9 (4.5) |
| Esophagus | 1 | 2.9 (4.5) | 1 | 2.9 (4.5) | 0 | 0 (0) |
| Subcutis | 1 | 2.9 (4.5) | 1 | 2.9 (4.5) | 0 | 0 (0) |
| Multiple organs | 11 | 31.4 (50) | 4 | 11.4 (18.2) | 7 | 20.0 (31.8) |
| Liver, lung | 5 | 14.3 (22.7) | 1 | 2.9 (4.5) | 4 | 11.4 (18.2) |
| Liver, lung, peritoneum | 1 | 2.9 (4.5) | 1 | 2.9 (4.5) | 0 | 0 (0) |
| Liver, lung, stomach | 1 | 2.9 (4.5) | 1 | 2.9 (4.5) | 0 | 0 (0) |
| Liver, lung, kidney | 1 | 2.9 (4.5) | 0 | 0 (0) | 1 | 2.9 (4.5) |
| Liver, spleen, lymph nodes | 1 | 2.9 (4.5) | 0 | 0 (0) | 1 | 2.9 (4.5) |
| Spleen, adrenal gland | 1 | 2.9 (4.5) | 0 | 0 (0) | 1 | 2.9 (4.5) |
| Lung, cerebrum, adrenal gland | 1 | 2.9 (4.5) | 1 | 2.9 (4.5) | 0 | 0 (0) |

Abbreviations: CRP, C-reactive protein
